# Volumetric modulated arc planning for lung stereotactic body radiotherapy using conventional and unflattened photon beams: a dosimetric comparison with 3D technique

**DOI:** 10.1186/1748-717X-6-152

**Published:** 2011-11-09

**Authors:** Geoffrey G Zhang, Lichung Ku, Thomas J Dilling, Craig W Stevens, Ray R Zhang, Weiqi Li, Vladimir Feygelman

**Affiliations:** 1 Radiation Oncology, Moffitt Cancer Center, Tampa, Florida, USA; 2 Radiation Oncology, Salem Hospital, Salem, Oregon, USA; 3 School of Medicine and Public Health, University of Wisconsin, Madison, Wisconsin, USA

## Abstract

**Purpose:**

Frequently, three-dimensional (3D) conformal beams are used in lung cancer stereotactic body radiotherapy (SBRT). Recently, volumetric modulated arc therapy (VMAT) was introduced as a new treatment modality. VMAT techniques shorten delivery time, reducing the possibility of intrafraction target motion. However dose distributions can be quite different from standard 3D therapy. This study quantifies those differences, with focus on VMAT plans using unflattened photon beams.

**Methods:**

A total of 15 lung cancer patients previously treated with 3D or VMAT SBRT were randomly selected. For each patient, non-coplanar 3D, coplanar and non-coplanar VMAT and flattening filter free VMAT (FFF-VMAT) plans were generated to meet the same objectives with 50 Gy covering 95% of the PTV. Two dynamic arcs were used in each VMAT plan. The couch was set at ± 5° to the 0° straight position for the two non-coplanar arcs. Pinnacle version 9.0 (Philips Radiation Oncology, Fitchburg WI) treatment planning system with VMAT capabilities was used. We analyzed the conformity index (CI), which is the ratio of the total volume receiving at least the prescription dose to the target volume receiving at least the prescription dose; the conformity number (CN) which is the ratio of the target coverage to CI; and the gradient index (GI) which is the ratio of the volume of 50% of the prescription isodose to the volume of the prescription isodose; as well as the V20, V5, and mean lung dose (MLD). Paired non-parametric analysis of variance tests with post-tests were performed to examine the statistical significance of the differences of the dosimetric indices.

**Results:**

Dosimetric indices CI, CN and MLD all show statistically significant improvement for all studied VMAT techniques compared with 3D plans (p < 0.05). V5 and V20 show statistically significant improvement for the FFF-VMAT plans compared with 3D (p < 0.001). GI is improved for the FFF-VMAT and the non-coplanar VMAT plans (p < 0.01 and p < 0.05 respectively) while the coplanar VMAT plans do not show significant difference compared to 3D plans. Dose to the target is typically more homogeneous in FFF-VMAT plans. FFF-VMAT plans require more monitor units than 3D or non-coplanar VMAT ones.

**Conclusion:**

Besides the advantage of faster delivery times, VMAT plans demonstrated better conformity to target, sharper dose fall-off in normal tissues and lower dose to normal lung than the 3D plans for lung SBRT. More monitor units are often required for FFF-VMAT plans.

## Introduction

Studies have shown encouraging results when treating medically inoperable early stage lung cancer using stereotactic body radiotherapy (SBRT) [1-3]. Conventionally, lung cancer SBRT has been delivered using three-dimensional (3D) non-coplanar beams [4] or IMRT. Recently, volumetric modulated arc therapy (VMAT) was introduced to treat various disease sites [5-7], including lung SBRT [8,9]. The major advantages of the VMAT SBRT plans compared to the conventional 3D ones include faster delivery, which reduces the risk of intrafractional motion, while simultaneously improving target dose conformity [8,9]. VMAT plans lead to a smaller percentage of lung volume exceeding 5 Gy, (V5), and 20 Gy, (V20) [10].

Recently, a flattening-filter-free (FFF) linear accelerator was installed in our clinic. As part of critical evaluation of this new technology, we included FFF-VMAT into comprehensive comparisons of the dosimetric parameters for different lung SBRT treatment techniques. We studied the dose conformity to the target volume, the dose fall-off in normal tissues, and the V20, V5, mean lung dose (MLD), mean PTV dose, as well as total monitor units (MU) for VMAT, FFF-VMAT and 3D conformal SBRT plans. In the present paper, we statistically compare these techniques.

## Materials and methods

### Treatment planning

This retrospective study was approved by the institutional IRB. A total of 15 early stage lung cancer patients with various tumor sizes previously treated with 3D or VMAT SBRT were randomly selected. Four-dimensional (4D) CT was used for each patient to determine the internal gross tumor volume (IGTV) to account for the respiratory motion. Abdominal compression was applied to thirteen patients to minimize respiratory excursion of the diaphragm. For two patients, no abdominal compression was applied, at the discretion of the treating radiation oncologist. A superior/inferior margin of 0.7 cm and axial margin of 0.5 cm was added to the IGTV to generate the planning target volume (PTV). The average PTV was 61.0 cm^3 ^(range 16.8-160.8 cm^3^). For each patient, we generated a non-coplanar 3D plan, both coplanar and a non-coplanar conventional VMAT plans, and a non-coplanar FFF-VMAT plan. The same dose objectives were used for each plan. They were designed to deliver 50 Gy in 5 fractions to 95% of the PTV. Two dynamic arcs were used in all VMAT plans. The couch was offset ± 5° for the non-coplanar arcs. Pinnacle version 9.0 (Philips Radiation Oncology, Fitchburg WI) was used to plan for a 6 MV or 6MV-FFF beams from a TrueBeam linear accelerator (Varian Medical System Inc. Palo Alto, CA). SmartArc was used for the VMAT planning [11]. All plans were designed to spare the contralateral lung as much as possible. To that end, no arc beam entrance through the uninvolved lung was allowed. Thus no full rotation arcs were used. The same beam entrance strategy was also used in the 3D plans, with 9-11 beams, of which 4-9 were non-coplanar. A 1 cm wide avoidance ring structure was used in plan optimization to facilitate rapid dose fall-off away from the PTV. The Pinnacle's direct machine parameters optimization (DMPO) feature was used in the beam weighting optimization by allowing one segment per beam for the 3D plans. Inhomogeneity correction was used in all the plans.

### Conformity index

The conformity index (CI) is defined as the ratio of the total volume receiving at least the prescription dose, *V*_100_, to the target volume receiving at least the prescription dose, *Vt*_100_:[12]

(1) $$CI=V_{100}∕Vt_{100}$$

The value of CI is always greater than unity. A value that is closer to unity represents a better target conformity of radiation dose in the treatment plan.

### Conformity number

The target coverage (TC) is defined as the ratio of the target volume receiving at least the prescription dose, *Vt*_100_, to the total target volume, *Vt*:

(2) $$TC=Vt_{100}∕Vt$$

The value of CI varies with the value of TC. Poor target coverage may give a better CI. To include the effect of TC on conformity, the conformity number (CN) is introduced and defined as the ratio of the TC to CI [13]:

(3) $$CN=TC/CI=Vt_{100}^{2}/\left(V_{100}\cdot Vt\right)$$

The value of CN is always smaller than unity. A value closer to unity represents a better conformity and target coverage.

### Gradient index

The gradient index (GI) is defined as the ratio of the volume covered by at least a given percentage of the prescription dose to the volume covered by the full prescription dose [14]. For this lung SBRT dosimetric study, the given percentage is set at 50% of the prescription dose. Mathematically, GI in this study is expressed as:

(4) $$GI=V_{50}∕V_{100}$$

where *V_50 _*is the volume covered by at least 50% of the prescription dose.

The value of GI is greater than unity. A value that is closer to unity represents a faster dose fall-off in normal tissue in the treatment plan, which may imply a lower dose to critical structures.

### V20, V5, and Mean Lung Dose

The percentage of normal lung volume exceeding 20 Gy of dose, V20, is a key parameter in risk assessment of radiation pneumonitis [15], and is often used in thoracic cancer treatment plan evaluation [1,16]. Other studies also found close correlation between high percentage normal lung volume exceeding 5 Gy of dose (V5) and pneumonitis [17,18]. Normal lung volume was defined as the total lung volume minus GTV. Our treatment planning objectives required a V20 < 10%. The mean lung dose (MLD) is another important index in radiation pneumonitis risk assessment [19]. The values of V20, V5 and MLD were compared between the planning methods for each case. The generalized equivalent uniform dose (gEUD) [20,21] was calculated for normal lung volume and compared between all the plans.

### Total monitor units

At distances beyond a few cm from the field edge, peripheral dose is dominated by accelerator head leakage and is therefore proportional to total MU. Monitor units in each plan were compared as a risk index for potential radiation-induced secondary malignancies [22]. The accelerators are calibrated to deliver 1 cGy/MU to muscle at a depth of maximum dose (1.5 cm) for a 10 × 10 field at source-to-surface distance of 100 cm.

### Mean dose in PTV

The mean target dose (MTD) was used to quantify dose homogeneity inside the PTV. With the same prescribed dose and target coverage, higher MTD implies a more heterogeneous dose distribution within the PTV. Additionally, the gEUD was calculated for the PTV based on the dose distribution and compared between all the plans.

### Statistical analysis

The non-parametric Friedman test was applied in the statistical analysis. The Friedman test compares three or more paired groups. A p value is generated by the Friedman test. If it is small (< 0.05), the null hypothesis that there is no difference between the column median values is rejected. The paired test is chosen because the underlying physical problem is identical across the planning techniques for each patient but varies among the patients.

Following the Friedman test, the rank-based multiple comparison test, Dunn's post-test, was performed. It tests the same null hypothesis for individual pairs of data columns. Dunn's post-test includes a non-parametric equivalent of Bonferroni adjustment for multiple comparisons.

## Results

Table 1 lists the statistical data of the dosimetric indices for all the studied cases. Overall, VMAT plans demonstrate better indices than the 3D plans. The non-coplanar VMAT plans yield slightly better indices than the coplanar ones. The GI values for the FFF-VMAT plans were slightly lower than those for conventional plans. VMAT required higher total MUs than 3D. The FFF-VMAT plans tended to use the highest number of MU.

**Table 1:** Dosimetric data of 3D, coplanar, non-coplanar VMAT and FFF-VMAT plans.

Plan	3D	Coplanar VMAT	Non-coplanar VMAT	Non-coplanar FFF-VMAT
CI	1.44 ± 0.21	1.23 ± 0.20	1.22 ± 0.17	1.19 ± 0.13

CN	0.67 ± 0.09	0.79 ± 0.09	0.79 ± 0.08	0.81 ± 0.07

GI	7.12 ± 1.43	6.49 ± 1.90	6.41 ± 1.78	6.23 ± 1.59

V20 (%)	6.8 ± 2.9	5.8 ± 2.4	5.8 ± 2.3	5.6 ± 2.3

V5 (%)	23.5 ± 8.0	20.9 ± 7.4	21.0 ± 7.2	20.4 ± 7.1

MLD (cGy)	497 ± 153	458 ± 144	459 ± 140	449 ± 142

MTD (cGy)	5359 ± 101	5370 ± 116	5361 ± 104	5324 ± 83

MU	1528 ± 136	1708 ± 194	1660 ± 200	1805 ± 258

In the table, CI = conformity index, CN = conformity number, GI = gradient index, V20 is the percentage volume of lung-GTV exceeding 20 Gy, V5 is the percentage volume of lung-GTV exceeding 5 Gy, MLD = mean lung dose, MTD = mean target dose and MU = number of monitor units.

Due to the large differences in PTV size as well as individual patient anatomical variations, the standard deviations in Table 1 are quite high. This large spread somewhat obscures the dosimetric differences between the plans. To better illuminate the differences, all the plans were compared with the corresponding 3D plans taken as a reference. The numbers of cases out of the 15 cases studied that dosimetrically favor the VMAT or FFF-VMAT plans are presented in Table 2. Except for the total MUs, more cases favor the VMAT and FFF-VMAT plans for all other dosimetric indices.

**Table 2:** Compared to 3D plans, number of cases that is dosimetrically in favor of the VMAT or FFF plans.

Index	CI	CN	GI	V5	V20	MLD	MTD	MU
Coplanar VMAT	14	14	12	12	13	15	5	0

Non-coplanar VMAT	14	14	12	13	14	14	8	2

Non-coplanar FFF-VMAT	15	15	13	14	15	14	11	0

The total number of cases is 15. In the table, CI = conformity index, CN = conformity number, GI = gradient index, V20 is the percentage volume of lung-GTV exceeding 20 Gy, V5 is the percentage volume of lung-GTV exceeding 5 Gy, MLD = mean lung dose, MTD = mean target dose and MU = number of monitor units.

### Conformity index (CI)

The overall Friedman test demonstrated highly significant difference between the techniques (p < 0.0001) for the CI. Individual comparisons (Dunn's post-test) for the CI indicate statistically significant difference between the 3D and all VMAT techniques: p < 0.05 between the 3D and the coplanar VMAT plans, p < 0.01 between 3D and the non-coplanar VMAT plans and p < 0.001 between 3D and the FFF-VMAT plans,. The differences were not significant (p > 0.05) between different VMAT plans. VMAT offers statistically significant improvement in CI over 3D.

### Conformity number (CN)

Similar to CI, the Friedman test indicated overall significant difference in CN (p < 0.0001). The p values of the Dunn's post-test were p < 0.05 between 3D and the coplanar VMAT plans, p < 0.01 between 3D and the non-coplanar VMAT plans and p < 0.001 between 3D and the FFF-VMAT plans. No significant difference in CN between VMAT techniques could be established (p > 0.05)

### Gradient index (GI)

The overall p value from the Friedman test was 0.0035 for GI. The Dunn's test yielded p < 0.05 between 3D and the non-coplanar VMAT plans and p < 0.01 between 3D and the FFF-VMAT plans. The test demonstrated no significant GI improvement (p > 0.05) for coplanar VMAT compared to 3D, and no significant differences among the different VMAT techniques.

### V20, V5 and mean lung dose (MLD)

From the overall Friedman test, p < 0.0001 for V20. A statistically significant improvement in V20 was found for non-coplanar VMAT (p < 0.01) and FFF-VMAT (p < 0.001) techniques compared to 3D. No significant difference in V20 was found between 3D and coplanar VMAT, as well as between different VMAT techniques.

The Friedman test yielded p = 0.0002 for V5 comparisons. Individual comparisons showed no statistically significant differences, except for the FFF-VMAT plans indicating improvement in V5 compared to 3D (p < 0.001).

The Friedman test was significant (p < 0.0001) for MLD comparisons. The Dunn's post test demonstrated that all VMAT techniques showed improvement in MLD compared to 3D with p < 0.05 between 3D and the coplanar VMAT, p < 0.01 between 3D and the non-coplanar VMAT and p < 0.001 between 3D and FFF-VMAT. No significant difference in MLD was shown between the VMAT techniques. The gEUDs for the normal lung closely followed the MLD.

### Monitor units (MU)

The overall Friedman test p value for the MU was p < 0.0001. The MUs were significantly different between FFF-VMAT and 3D (p < 0.001) and between FFF-VMAT and non-coplanar VMAT (p < 0.05). There was also a difference between 3D and coplanar VMAT (p < 0.001). The MU in the FFF-VMAT plans was always greater than in 3D plans.

### Mean dose in PTV (MTD)

The average MTD was lowest in the FFF-VMAT plans. The p value from the Friedman test for MTD was 0.0052. The Dunn's test only showed significant difference between the coplanar VMAT and FFF-VMAT techniques (p < 0.01). These are the planning techniques with the highest and lowest average MTD values. The comparisons of the PTV gEUD closely followed the MTD comparisons.

## Discussion

The VMAT advantage in shortening the treatment time compared to 3D non-coplanar plans is well known [9]. The current study also shows that VMAT plans are more likely to give statistically better target conformity and sharper dose fall-off in normal tissues. The average GI value for FFF-VMAT plans was lower than that for 3D plans, indicating better dose fall-off in normal tissues in the FFF-VMAT plans. At the same time, V20 and V5 were lower when treating with FFF-VMAT. V20 was also lower in non-coplanar VMAT plans than in 3D.

In general, the comparison of V20, V5 and MLD between VMAT and 3D in this study agrees with the study by McGrath *et al *[23], but not with Chan *et al *[24]. In Chan *et al*, the comparison was made for the treatment of the locally advanced non-small cell lung cancer. The target volume was usually larger than is suitable for SBRT. In their comparison, mean lung dose and V20 were similar between 3D and VMAT, but V5 was significantly higher in VMAT. The comparison of other parameters in Chan *et al*, such as CI and MU, agree with our study.

Due to collision limitations, the angle separation of the non-coplanar arcs was uniformly set at 10° (± 5° to the straight couch position). It is plausible that the dosimetric differences between the coplanar and non-coplanar VMAT plans were not statistically significant due to this small physical angle separation as opposed to the limited statistical power. We further postulate that with a larger angle separation, the differences are expected to increase, favoring the non-coplanar VMAT plans for the CI, GI and V20 values. The angular separation can be larger for smaller patients or when the tumor is situated more laterally. The other way to increase the angle separation is to shift the isocenter laterally, away from the tumor. We have not explored this option.

**Figure 1:**
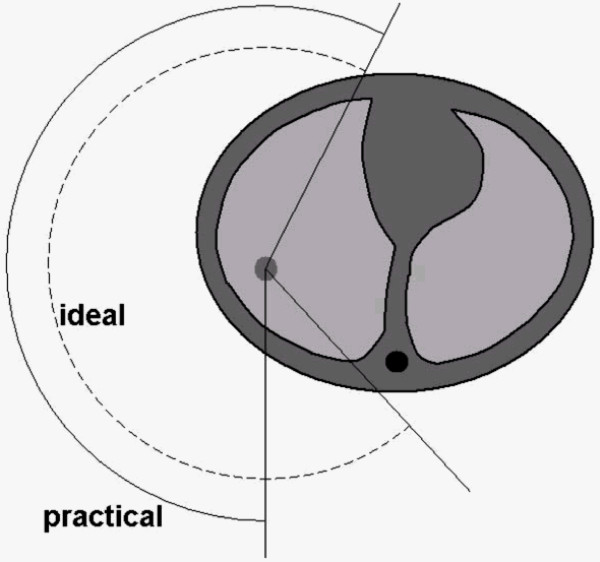
**Due to the limitation that the gantry of a Varian accelerator cannot pass from 180° to -180° and vice versa, the ideal arcs (dashed arc in this example) often have to be shortened to be deliverable (solid arc)**.

Beam arrangements are more restrictive in VMAT planning compared to 3D conformal planning. First, the couch angle range for non-coplanar planning is smaller with the arcs compared to the static beams. Second, the machine limitation prohibiting the gantry from crossing the 180 degree position often shortens the arc length in VMAT planning, as shown in Figure 1. This could affect the quality of the VMAT plans.

If increased dose heterogeneity is accepted within the target volume, dose fall-off can be sharpened in normal tissue [25]. Because FFF-VMAT plans tend to deliver a more homogeneous dose to the target volumes, the GI should be improved if the dose constraints mandate a higher mean dose in the targets. The other factor that affects the dose fall-off in normal tissue is the beam aperture margin. In SmartArc planning, this margin is not fixed, but is rather optimized by the software. An option to allow user to use a fixed margin with the arcs would help to improve the dose fall-off in normal tissue.

For off-center PTV volumes, FFF-VMAT typically requires more MU due to the highly peaked beam profile. This does not automatically translate into higher peripheral doses as there is less head scatter and leakage from an accelerator without a flattening filter [26].

The dose rate for the FFF beams can be substantially higher than the conventional beams (1400 or 2400 MU/min vs. 600-1000 MU/min). However, for VMAT plans, the treatment delivery time is largely limited by the gantry rotation speed, not the dose rate.

The interplay between the dynamic MLC-based delivery of VMAT and the respiratory motion of the tumor may degrade target coverage [27]. Since this is not a concern with 3D technique, we expect a better agreement in target coverage between the plan and delivered treatment for 3D treatment compared to VMAT if there is significant respiratory tumor motion or if the beam aperture size is frequently small in a VMAT plan. We are currently exploring the impact of tumor motion on target coverage when using IMRT technique.

## Conclusions

CI, CN and mean lung dose are highly statistically improved for all studied VMAT techniques compared with 3D plans. GI, V5 and V20 are statistically improved for FFF-VMAT plans compared with 3D technique. It is also clear that FFF-VMAT plans require more monitor units than 3D or non-coplanar VMAT techniques.

## Competing interests

The authors declare that they have no competing interests.

## Authors' contributions

GZ: initiated and organized the project; performed the bulk of data collection and analysis; prepared the draft of the manuscript. LK: participated in the design of the study and the draft of the manuscript. TD: contoured PTVs and provided prescriptions; monitored treatment planning; contributed to the draft of the manuscript. CS: contoured PTVs and provided prescriptions; monitored treatment planning; contributed to the draft of the manuscript. RZ: contributed to the data analysis and the draft of the manuscript. WL: generated most of the treatment plans. VF: supervised the project; contributed to the statistical data analysis and the draft of the manuscript; participated in its design and coordination. All authors read and approved the final manuscript.
